# High inter-observer agreement of observer-perceived pain assessment in the emergency department

**DOI:** 10.1186/s12873-018-0159-4

**Published:** 2018-02-21

**Authors:** Martin Høhrmann Hangaard, Brian Malling, Christian Backer Mogensen

**Affiliations:** 1 Upper GI Section, Department of Surgery, Odense University Hospital, Sdr. Boulevard 29, DK-5000 Odense C, Denmark; 2 Department of Diagnostic Radiology, Rigshospitalet, Blegdamsvej 9, DK-2100 København Ø, Denmark; 3 Emergency Department, Hospital of Southern Jutland, Kresten Philipsensvej 15, DK-6200 Aabenraa, Denmark

## Abstract

**Background:**

Triage is used to prioritize the patients in the emergency department. The majority of the triage systems include the patients’ pain score to assess their level of acuity by using a combination of patient reported pain and observer-perceived pain; the latter therefore requires a certain degree of inter-observer agreement.

The aim of the present study was to assess the inter-observer agreement of perceived pain among emergency department nurses and to evaluate if it was influenced by predetermined factors like age and gender.

**Method:**

A project assistant randomly recruited two nurses, who were not allowed to interact with each other, to assess patient pain intensity on the numeric ranking scale. The project assistant afterwards entered the pain scores in a predesigned electronic questionnaire.

We used weighted Fleiss-Cohen (quadratic) kappa statistics, Bland-Altman statistics and logistic regression analysis to assess the inter-observer agreement.

**Results:**

One hundred and sixty-two patients were included. They had a median age of 38 years and 45% were females. 30% of the patients were acute surgical patients and 70% acute orthopedic patients. The average time between the pain assessments were 1,7 min. The Bland Altman analysis found a mean difference in pain score of 0.2 and 95% limits of agreement of +/− 3 point. When the NRS scores were translated to commonly used pain categories (no, mild, moderate or severe pain) we found a 70% agreement with a mean difference in categories of 0.05 and 95% limits of agreement of +/− 1 category. Patient age, gender, localization of pain, examination room or presence of a significant other did not affect the inter-observer agreement.

**Conclusion:**

We found 70% agreement on pain category between the nurses and it is justified that nurse-perceived pain assessment is used for triage in the emergency department.

## Background

Pain is one of the most common complaints in the emergency department (ED) [1]. Primarily a subjective perception, it is not easily quantified and depends on various factors, including age, gender, surroundings, and previous experience with pain [2, 3]. Previous studies have found that nurses tend to underestimate patients’ pain intensity [4–6]. The discrepancy between the patient’s self-reported pain and nurses’ estimations is higher when assessing abdominal pain compared to bone fracture pain [4] and in younger than older patients [7, 8].

Pain assessment is an integrated part of many triage systems, which determine the patient’s level of acuity and, thus, their treatment priority. Using patient-reported pain assessments creates risks of misclassification, either as over-triage, which binds resources unnecessarily, or under-triage, where a serious condition risks going unrecognized.

Some triage systems have tried to compensate for this risk of misclassification by using observer-perceived pain assessment, either instead of or in combination with the patient-reported pain assessment. In the Danish Emergency Process Triage (DEPT), which has been implemented in most Danish EDs [9], patient-reported pain is validated by a nurse to ensure that it is neither over nor underreported, resulting in a patient receiving a higher triage priority than warranted or, alternatively, that a patient might be overlooked in the ED. [10]

However, observer-perceived pain assessments in the triage process require a certain level of agreement between ED nurses when rating patient pain. Thus far, no studies have examined the inter-observer agreement of perceived pain among nurses in an ED setting, which is the primary aim of this study. As a secondary aim we assessed whether patient location (emergency room or a ward room), type of pain or the presence of a significant other would affect this inter-observer agreement.

## Methods

This was a cross-sectional, single-center study conducted at the ED in the Hospital of Southern Jutland, Denmark, between October 23 and November 26, 2013. The ED consisted of “emergency rooms” equipped with facilities for resuscitations and surgical procedures and “ward rooms” with only a hospital bed and facilities for monitoring.

All of the patients with musculoskeletal pain or abdominal pain assessed in the ED were invited to participate in the study if they were able to speak Danish, German, or English. Patients with obvious life- or limb-threatening conditions and patients who were unable to provide informed consent were excluded, as were patients with no pain complaint. We did not include patients with chest pain, as this group always receives high-level triage according to most triage systems, including the DEPT.

Two project assistants identified the patients who met the inclusion criteria and received the informed consent to participate. All of the ED nurses participated in the study. Among the staff on duty, two available nurses were recruited to assess patient pain intensity on the numeric ranking scale (NRS) in the same way that they would normally according to the DEPT instructions. The first nurse went to the patient, secured privacy, and asked them to assess their pain on a 10-point NRS scale. The nurse then assessed the patient’s pain on the same scale without informing the patient of the result, went out, and immediately reported the patient’s score and the nurse score to the project assistant.

The second nurse then went to the patient and performed the same procedure. The nurses were not allowed at any time to discuss or compare their results with each other. The project assistants entered the scores into a predesigned electronic questionnaire together with the time, patient age, gender, main complaint, the presence of a significant other, if the patients had received analgesics, and if the pain assessment was in an emergency room or a ward room.

The data was collected on random days, including weekends, from 9 am to 6 pm, which was the time with the highest patient flow.

The study was powered to detect a mean difference of 2 NRS points between observers, giving a sample size of 44 paired observations, based on a power of 90% and a confidence interval of 95%. For the kappa statistics, a kappa value of 0.60 was expected and the study was powered to detect a difference in positive proportion in a dichotomous test between observers of 10% with a 95% confidence interval not larger than kappa +/− 0.11, resulting in a sample size of 164 patients.

The data from the electronic questionnaire was transferred to Stata 14 for statistical analysis.

We used weighted Fleiss-Cohen (quadratic) kappa statistics, which accounts for a situation where there were constantly two raters but their identity varied and the rating was in ordered categories. We also calculated the Bland-Altman statistics to assess the agreement between the two rating nurses. We then transferred the NRS assessment to four categories of pain in accordance with the definitions in the DEPT: No pain 0, mild pain as NRS 1–3; moderate pain as 4–6 and severe pain as 7–10 [11] and repeated the same statistical analysis. Finally, we performed a logistic regression analysis to identify if gender, age, location of pain in the ED, and presence of significant other would influence the inter-observer agreement.

Since the study only involved an assessment of the patients’ pain, no acceptance from an ethical committee was required according to Danish legislation. The study was registered with the Danish Data Protection Agency.

## Results

A total of 163 patients participated in the study. One patient received analgesics between the nurses’ respective assessments and was therefore excluded. The remaining 162 patients had a median age of 38 years (p25–p75: 20–61 years) and 73 were females (45%), 48 of the patients (30%) had abdominal pain and 114 (70%) musculoskeletal pain. The mean time between the two pain assessments was 1.7 min (95% CI: 1.2–2.3 min). The data was primarily collected during working days (76%). The inclusion time was from 9 am to 6 pm, with 57% of the inclusions performed before 2 pm.

**Fig. 1:**
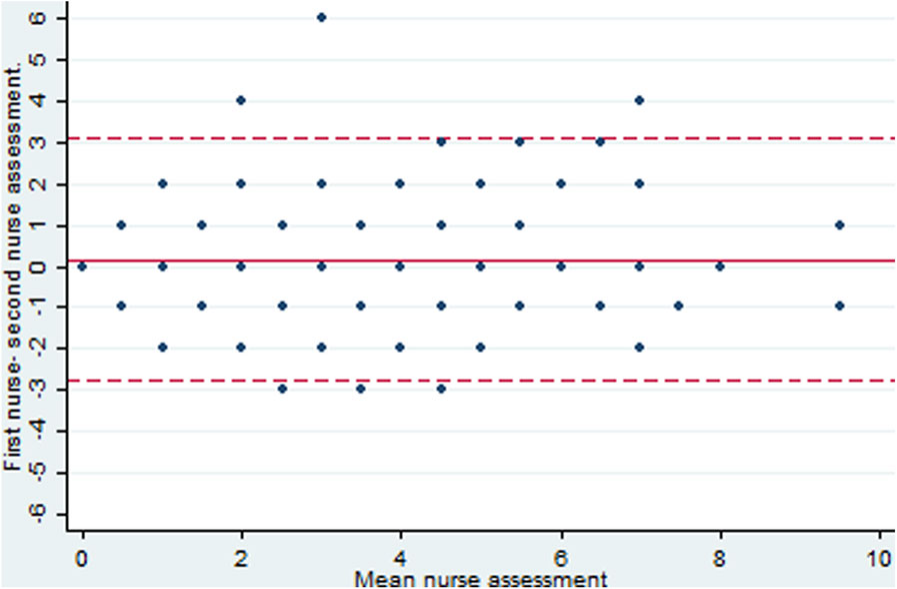
Bland-Altman plot for NRS assessment

The mean NRS scores between Nurse 1 (3.8, 95% CI: 3.4–4.1) and Nurse 2 (3.6, 95% CI: 3.3–3.9) did not differ significantly (p: 0.18), with a Kappa-value of 0.55 (95% CI: 0.48–0.57), i.e. a moderate agreement [12]. The Bland Altman analysis revealed that the mean difference in pain score was 0.2 (95% CI: -0.1–0.4), and the 95% prediction interval for the Nurse 2 score was from − 2.8–3.1 on the NRS scale, i.e. the second nurse would score within 3 points higher or lower compared to Nurse 1. This is reflected in the Bland-Altman plot in Fig. 1.

**Fig. 2:**
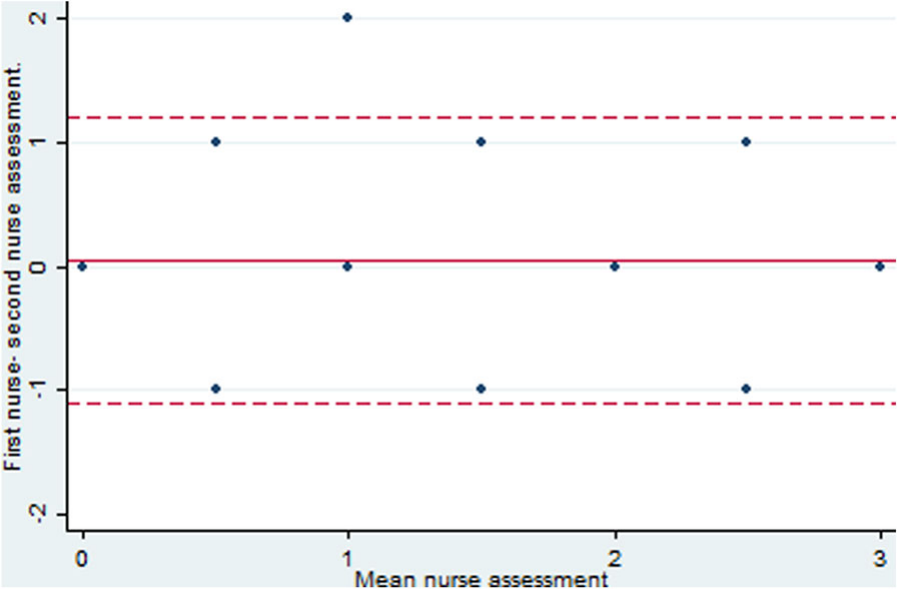
Bland-Altman plot for pain categories

Table 1 shows the distribution of pain assessments in the categories between the two nurse observations of the same patient. There was an overall agreement of 70% (95% CI: 63–77%), with a Kappa-value of 0.55 (0.51–0.65), moderate agreement. The difference between the nurses was greater than one category for only two of the patients (1%). The Bland-Altman analysis showed a mean difference of 0.05 categories with a 95% prediction interval of +/1 category (Fig. 2).

**Table 1:** Two nurses perceptions of 162 patients pain categories

Second nurse pain assessment						
		no	mild	moderate	severe	Total
First nurse pain assessment	no	1	3	0	0	4
	mild	2	60	15	0	77
	moderate	2	15	47	3	67
	severe	0	0	8	6	14
Total		5	78	70	9	162

Table 2 reflects the inter-observer agreement depending on age, gender, pain localization, examination room, and the presence of a significant other during the assessment. The only difference was a significantly higher agreement between the nurses among the patients aged 61–80 (OR 2.9, 95% CI 1.0–8.4), but this was not found after adjustments.

**Table 2:** Variables influencing the agreement among nurses in percieved pain categories of 162 patients

			univariat analysis		multivariat analysis (1)	
Variable	no. of agreements	% of agreements	OR	95% CI	OR	95% CI
gender						
female	50	68%	1			
male	64	72%	1.2	0.6-2.3		
age group						
< 20 yrs	23	58%	1			
20-40 yrs	35	74%	2.1	0.9-5.4	2.0	0.6-7.1
41-60 yrs	24	75%	2.2	0.8-6.1		
61-80 yrs	28	80%	2.9	1.0-8.4		
> = 80 yrs	4	50%	0.7	0.2-3.4		
specialty						
surgical	37	77%	1			
ortopaedic	77	68%	0.6	0.3-1.4		
significant other						
not present	61	76%	1			
present	53	65%	0.6	0.3-1.1		
room						
emergency room	80	68%	1			
ward room	34	77%	1.6	0.7-3.6		

(1) adjustment for gender, age, specialty, relative present and examination room

## Discussion

We found that when two nurses independently assessed the same patient, the inter-observer agreement had a kappa value of 0.55, which correlates to a moderate agreement.

Transferred to commonly used pain categories of no, mild, moderate or severe pain, a 70% agreement among the nurses was found, with a 95% prediction interval of +/− 1 pain category. This was independent of age, gender, presence or absence of a significant other, whether the patient was assessed in an emergency room or ward room, or if the patient complained of abdominal or musculoskeletal pain. The nurses were disagreeing more than one out of four pain categories in only 1% of the assessments.

Few studies have compared the inter-observer variation of pain assessment among nurses, and none of these studies have been in emergency departments. We are thus unable to compare our results directly with others in similar work environments. Studies based on theoretical pain cases revealed that there was a certain variation between nurses’ assessments, which could be attributed to the patient’s age, type and stage of illness, as well as the nurses’ professional experience and personal experiences of pain [13, 14]. Furthermore, pain assessment tools have been developed and validated for pain assessment in patients who are unable to express their sense of pain due to dementia or other mental distortions. The validation of these tools showed an acceptable agreement between the nurses [15, 16].

Our results have some clinical implications. The inter-observer agreement was lower than we had expected in a group of experienced ED nurses, who were well trained in using the NRS scale, but we found high inter-observer agreement when pain scores were transferred to commonly used pain categories. Also, the assessment of patient pain intensity where not influenced by different patient groups, age, gender, or other circumstances. Thus, our results justifies that nurse-perceived pain assessment is used for triage in the emergency department.

The advantage of our study was that it is the first to evaluate the inter-observer agreement of perceived pain of patients in an ED setting and the study has a reasonable size. However, the study is weakened by a number of factors. It was performed in an ED where all of the nurses had been trained in the use of NRS scales and had a considerable clinical ED experience, on average more than 2 years. A more heterogeneous nursing staff might have produced different results. Furthermore, we did not include patients with chest pain, and our results should not be extended to this group of patients. Moreover, we did not include patients who did not complain about pain. This might be considered as a bias, but for the evaluation of the clinical use of a triage system, which uses pain as a variable, it does not make sense to assess patients who were not complaining of pain or had no pain-associated reason for referral to the ED. Finally, since the nurses first asked the patients for their own pain assessment and first made their own assessment thereafter, the pain score was not absolutely independent. As this is how the currently used DEPT and other triage systems are used, our study reflects the pragmatic usage of the triage.

## Conclusion

This is the first study to validate inter-observer pain agreement in an emergency department. We found 70% agreement on pain category between the nurses, and the pain assessment was unaffected by age, gender, localization of pain, examination room, or whether or not a significant other was present. This justifies that nurse-perceived pain assessment is used for triage in the emergency department.

## Abbreviations

DEPT: Danish Emergency Process Triage
ED: Emergency Departments
NRS: Numeric Ranking Scale
